# Barriers and facilitators of people living with HIV receiving optimal care for hypertension and diabetes in Tanzania: a qualitative study with healthcare professionals and people living with HIV

**DOI:** 10.1186/s12889-023-17069-6

**Published:** 2023-11-13

**Authors:** Tiffany E. Gooden, Mkhoi L. Mkhoi, Mwajuma Mdoe, Lusajo J. Mwalukunga, Elizabeth Senkoro, Stephen M. Kibusi, G Neil Thomas, Krishnarajah Nirantharakumar, Semira Manaseki-Holland, Sheila Greenfield

**Affiliations:** 1 Institute of Applied Health Research, University of Birmingham, Birmingham, UK; 2 Department of Microbiology and Parasitology, University of Dodoma, Dodoma, Tanzania; 3 Department of Public Health, University of Dodoma, Dodoma, Tanzania; 4 Ifakara Health Institute, Ifakara, Tanzania

**Keywords:** Quality care, Patient safety, Prevention, Early diagnosis, Healthcare delivery

## Abstract

**Background:**

People living with HIV (PLWH) are at a higher risk for developing diabetes and hypertension. Often services are separate for HIV and non-communicable diseases (NCDs), but how this impacts NCD care among PLWH is unknown. We aimed to understand the barriers and facilitators for prevention, early diagnosis and safe effective care for diabetes and hypertension among PLWH.

**Methods:**

Semi-structured interviews (SSIs) were conducted with 10 healthcare professionals (HCPs) that care for PLWH, 10 HCPs that care for people with diabetes and hypertension and 16 PLWH with a comorbidity of diabetes and/or hypertension. Participants were recruited from two healthcare facilities in Dodoma, Tanzania and purposively sampled based on age and sex. Interviews were conducted in Swahili using pre-developed topic guides, audio recorded then translated verbatim into English. An inductive thematic analysis was conducted using The Framework Method.

**Results:**

Three themes were found: organisational/healthcare system factors, individual factors and syndemic factors. Organisational/healthcare system factors comprised the only facilitators for prevention (education on lifestyle behaviours and counselling on adherence), but included the most barriers overall: fragmented services, no protocol for NCD screening and lack of access to diagnostic equipment were barriers for early diagnosis whereas the former plus lack of continuity of NCD care were barriers for safe effective care. Individual factors comprised four sub-themes, three of which were considered facilitators: HCPs’ knowledge of NCDs for early diagnosis, self-monitoring of NCDs for safe effective care and HCPs’ personal practice for both early diagnosis and safe effective care. HCPs’ knowledge was simultaneously a barrier for prevention and PLWH knowledge was a barrier for prevention and safe effective care. Syndemic factors comprised three sub-themes; all were barriers for prevention, early diagnosis and/or safe effective care: poverty and mental health of PLWH and HIV stigma.

**Conclusions:**

Organisational/healthcare system, individual and syndemic factors were found to be interlinked with barriers and facilitators that contribute to the prevention, early diagnosis and safe effective care of diabetes and hypertension among PLWH in Tanzania; these findings can inform future initiatives for making small and large health system changes to improve the health of aging PLWH.

## Introduction

In 2019, there were 36.8 million people living with HIV (PLWH) worldwide, of which 71% reside in sub-Saharan Africa [1]. PLWH are at a higher risk of developing non-communicable diseases (NCDs) compared to people without HIV [2]. This is in part due to the toxicity of antiretrovirals (ARVs), microbial translocation, and persistent inflammation and immune response irrespective of viral load [3, 4]. However, the risk and burden of NCDs among PLWH may be heightened in sub-Saharan Africa due to the use of older and more toxic ARVs, elevated psychosocial factors related to poverty and low adherence to or interrupted use of ARVs which lead to irregularities of CD-4 counts and viral load, or a combination of these factors [3, 5, 6]. With increased access to effective ARVs and a reduction in deaths among PLWH globally [1], prevalence of HIV will continue to rise along with the incidence of NCD comorbidities. Thus, how to optimise NCD care for PLWH will increasingly be a priority worldwide, but particularly in sub-Saharan Africa where NCD and HIV burden is highest [1, 7]. Diabetes and hypertension are two NCDs that have gained particular attention due the aging of PLWH and the increased risk in incidence and high burden reported among PLWH.

For PLWH retained in care, visits for HIV care are routine with at least two visits a year [8], providing an opportunity for PLWH to be educated regarding prevention, signs and symptoms and to be screened for diabetes and hypertension based on recommendations and guidelines. In addition to traditional prevention strategies, early initiation and adherence to ARVs is key for PLWH [3, 6]. As per the general population, early diagnosis of diabetes and hypertension is imperative for a good prognosis; both conditions can partially be controlled through lifestyle modifications [9] and highly effective medication exist for reducing the risk of complications, development of additional comorbidities and premature mortality [10]. Once diagnosed, safe and effective care of diabetes and hypertension among PLWH is crucial for the conditions to be controlled and reduce risk of further morbidity. PLWH with diabetes or hypertension have special health needs that require continuity of care and careful consideration when prescribing medications to ensure patient safety, increase drug adherence and reduce drug-drug interactions and polypharmacy. Globally, care for NCDs and HIV are generally provided by separate physicians and often in different healthcare facilities, creating challenges for continuity of care and oversight of medications. The World Health Organisation (WHO) recommends the integration of HIV, diabetes and hypertension care to overcome such challenges [11]; however, changes to health services and systems, especially in low- and middle-income countries (LMICs) where resources are limited, will take time. Thus, understanding the barriers, facilitators and gaps of how the current approach of healthcare delivery for diabetes and hypertension for PLWH is important to understand.

Qualitative methods [12] enable an in-depth and contextual understanding regarding the complexities of healthcare delivery. A 2021 systematic review of qualitative studies on PLWH’s experiences of NCD comorbidities identified 14 qualitative studies; however, half (*n*= 7) were conducted in North America and five were conducted in South Africa [13]. Of the remaining two studies, one was conducted in Malawi and focused on PLWH with hypertension [14], and one was conducted in Ghana and included women with multimorbidity (many of whom were living with HIV) [15]. Evidence is therefore limited on PLWH’s experiences of diabetes and hypertension care from a low-income African setting. Tanzania is a lower-middle-income country [16] located in East Africa with a HIV prevalence of 4.5% [17]. An estimated 29% of PLWH have hypertension [18] and 13% have diabetes [19] in Tanzania compared to 17% [20] and 6% [21] in the general population, respectively. A recent (2021) qualitative study conducted in Eastern Tanzania investigated factors related to integration of NCD care, from the perspective of PLWH and healthcare professionals (HCPs) providing HIV care; most PLWH in the study had diabetes and/or hypertension [22]; however, it is vital to first have an in-depth understanding of any barriers and facilitators to optimal care with the current model of healthcare delivery to inform both short and long-term initiatives to improve services and reduce the burden of and improve optimal care for diabetes and hypertension among PLWH across sub-Saharan Africa. We aimed to understand the barriers and facilitators for prevention, early diagnosis and safe effective care for diabetes and hypertension within the current model of healthcare delivery among PLWH in Central Tanzania.

## Methods

### Study design

A pragmatic qualitative study [23] was conducted using semi-structured interviews [24] between 21^st^ October 2022 and 30^th^November 2022, combining thematic analysis with a descriptive phenomenology approach [25]. Qualitative methods [12] were used to enable HCPs and PLWH to openly express their experiences and perspectives of the current delivery of care for HIV, diabetes and hypertension and capture the in-depth and contextual factors related to any barriers and facilitators from their lived experiences. We chose to focus on diabetes and hypertension given the high burden [18] and increased risk [2] among PLWH, they can be clinical managed relatively easily, and the WHO recommends integrating HIV, diabetes and hypertension care [11] despite limited evidence on current barriers and facilitators for care.

### Study setting

This study was conducted in Dodoma, Tanzania, the country’s capital city located in the Central region with a population of around 760,000 people; however, over three million people reside in the greater Dodoma area which is mostly rural [26]. Participants were recruited from Dodoma Regional Referral Hospital and Makole Health Centre located within Dodoma city centre; the two combined see the largest number of PLWH in the Dodoma region (over 9000 PLWH in care at the time of data collection) and has the largest catchment area in Dodoma. Both facilities have a care and treatment centre (CTC) which provides care for HIV and an outpatient department (OPD) that provides care for NCDs, including diabetes and hypertension. The CTC system is not integrated with the OPD; PLWH with NCD comorbidities have a file within the OPD and a separate file at the CTC. ARVs, medication for opportunistic infections and blood tests (e.g. for checking viral load and CD-4 count) are provided free of cost to all PLWH within the CTC. Tests and medication for diabetes and hypertension are provided in the OPD, free of cost to those with insurance. PLWH are requested to attend the internal medicine outpatient clinic within the OPD for diabetes and hypertension once a month for follow-up visits with the clinician and prescription refills. Follow-up visits for HIV care are individualised based on a differentiated service delivery system where PLWH are categorised as stable or unstable based on viral load suppression and adherence to ARVs. PLWH with undetectable viral load and good adherence with at least six months since ARV initiation are categorised as stable and offered ARVs on a six-monthly cycle.

### Study participants

We interviewed HCPs working in the CTC, HCPs working in the OPD and PLWH with a comorbidity of diabetes and/or hypertension. The head ARV nurse at each facility recruited participants and were financially compensated for their efforts. PLWH were eligible if they were 18 years or older and had a comorbidity of diabetes and/or hypertension diagnosed after HIV between five years and three months prior to the interview to reduce recall bias regarding their NCD diagnosis, but with enough experience of receiving NCD care to ontribute to the study. CTC HCPs were eligible if they directly cared for PLWH and OPD HCPs were eligible if they directly cared for patients with diabetes or hypertension; both doctors and nurses were eligible if they had at least six months of work experience.

The head ARV nurses were asked to purposively recruit participants based on age and sex [27]. For PLWH, it was aimed to recruit at least half with hypertension and at least half with diabetes. Research staff visited the CTC on non-clinic days to interview HCPs. PLWH were recruited by phone at the Dodoma Regional Referral Hospital and in person at Makole Health Centre; participants from the former were asked to meet the data collectors at the CTC at a date and time that suited them whereas participants from the latter were interviewed either before or after their visit with the clinician. We aimed to reach data saturation with at least 10 CTC HCPs, 10 OPD HCPs and 16 PLWH [28, 29], with each participant group split evenly across the two hospitals.

### Data collection

Topic guides were developed in English then translated in Swahili. The topic guide for HCPs was developed based on the Theoretical Domains Framework [30] to understand how future changes to care could be successfully implemented (manuscript under review); however, the first two questions of the topic guide asked about barriers and facilitators for prevention, diagnosis, care and management of hypertension and diabetes for PLWH to enable an open discourse on their lived experiences regarding the current model of healthcare delivery. The remaining questions covered knowledge, skills, professional role, beliefs, decision processes, environmental context, resources and social influences regarding the care and management of PLWH with a comorbidity of hypertension and/or diabetes [30]. The topic guide for PLWH also included initial questions regarding barriers and facilitators for receiving a diagnosis and care for diabetes and/or hypertension; these were followed by questions about their experiences of diabetes and hypertension, pathways of care and healthcare seeking behaviours before and after their diabetes and/or hypertension diagnosis. Topic guides were finalised following review from and discussions with collaborators at the University of Dodoma to assess the questions for culturally and contextually appropriate wording, terminology and theory.

Interviews were conducted in private rooms within the CTC, audio recorded and facilitated by one of the two research assistants (MM and LJM) in Swahili. MM (female) and LJM (male) are both Tanzanian and are current PhD students at the University of Dodoma in Postpartum Care and Sexual and Reproductive Health, respectively; both have experience in qualitative methods and were trained on data collection for the current study. MM has a degree in midwifery and LJM has a degree in nursing; neither had a prior relationship with any of the participants. TEG, a female PhD candidate and research fellow in global health at the University of Birmingham, was present for half of the interviews (split across each participant group and interviewer to reduce interviewer effect) as an observer only; this work forms a chapter of her PhD thesis.

PLWH and HCPs were financially compensated for their participation (in Tanzanian Shillings equivalent to 2 USD and 10 USD, respectively); participants were unaware of compensation at the time of recruitment to avoid bias and undue influence [31]. On average interviews took around 40 minutes. Due to limited resources and time, interviews were transcribed directly into English by the bilingual research assistants that carried out the interviews; TEG created the English transcripts as MM and LJM verbally translated each audio recording verbatim. MM and LJM are both Tanzanian and was therefore able to concurrently clarify any contextual or cultural uncertainties for later interpretation.

### Analysis

To determine the patterns of barriers and facilitators related to the lived experiences of those providing and receiving care for HIV, diabetes and/or hypertension, an inductive thematic analysis was conducted using The Framework Method [32] after the completion of all interviews. Reading each transcript line-by-line, TEG manually analysed the data [33] by first applying open coding then forming a matrix of codes in Excel; this was completed separately for HCPs and PLWH. Categories were formulated through an iterative process of grouping and regrouping the codes, followed by the formulation of sub-themes that fed into the main themes. A qualitative expert and professor in medical sociology from the University of Birmingham (SG) independently coded the first four HCP transcripts to compare and check for completeness of codes and interpretation of the data. Themes and sub-themes were formulated directly from all data within the transcripts. Final themes and sub-themes were agreed following discussions with supervisors of the project (SG, GNT, KN, SMH) and the researchers that conducted the interviews (MM and LJM). All authors reviewed the themes and sub-themes with the opportunity to provide feedback and contribute to the interpretation of findings.

## Results

Interviews were conducted with 10 CTC HCPs (6 doctors, 4 nurses), 10 OPD HCPs (8 doctors, 2 nurses) and 16 PLWH (4 with diabetes, 8 with hypertension, 4 with diabetes and hypertension). Following transcription and translation of data, saturation was confirmed by the ninth interview with CTC HCPs, the eighth interview with OPD HCPs and by the thirteenth interview with PLWH; however, all data was used for the final analysis. CTC and OPD HCPs were evenly split across the two healthcare facilities, but only six PLWH were recruited from Makole Health Centre due to difficulties in recruitment. To avoid identification, only aggregate data of participant characteristics are provided in Table 1.

**Table 1:** Characteristics of participants: healthcare professionals (*n* = 20) and people living with HIV (*n* = 16)

Characteristics	CTC HCPs *N* = 10	OPD HCPs *N* = 10	PLWH *N* = 16
Sex			
Male	4	4	6
Female	6	6	10
Age			
20–30	4	1	0
31–40	1	6	0
41–50	2	1	3
51–60	2	2	8
61 +	1	0	5
Highest level of education			
No formal education	0	0	3
Primary education	0	0	11
Secondary education	0	0	1
University degree	10	10	1
Profession			
Doctor	6	8	–
Nurse	4	2	–
Duration of experience			
< 1 year	3	4	–
–10 years	5	4	–
> 10 years	2	2	–
Comorbidity			
Diabetes	–	–	4
Hypertension	–	–	8
Diabetes and hypertension	–	–	4

A dash indicates data that was not collected or not relevant to collect.

**Fig. 1:**
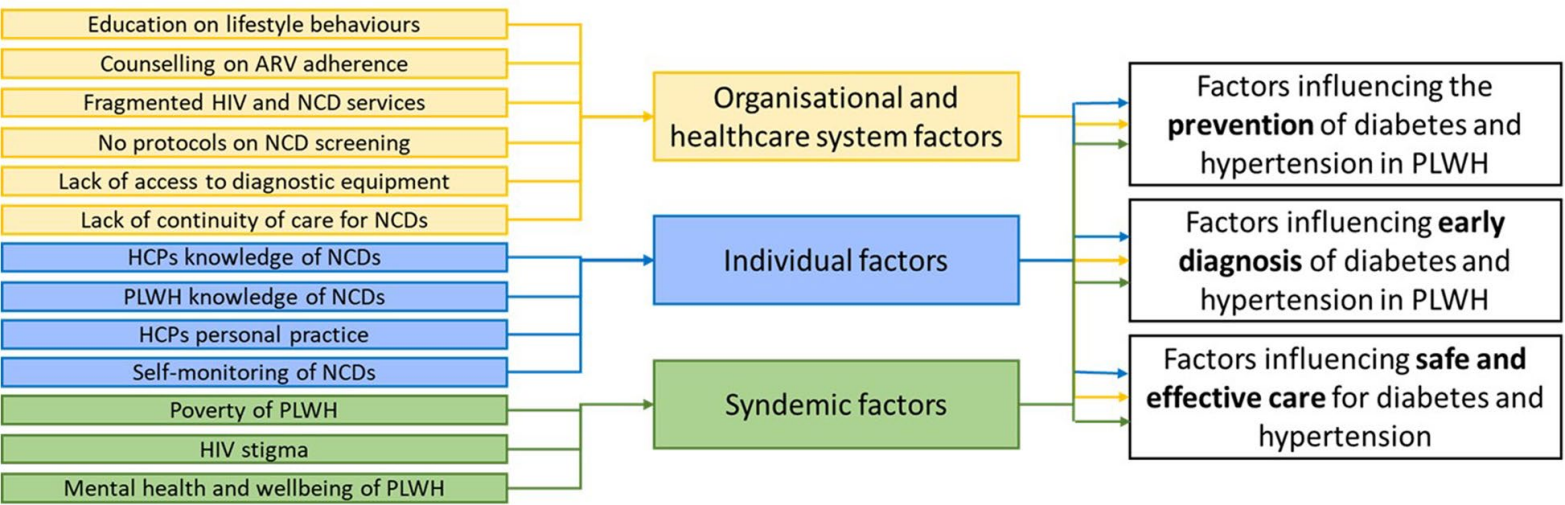
Coding tree of sub-themes that formed the main themes of which influence prevention, early diagnosis and safe effective care for diabetes and hypertension in PLWH

Three overarching themes were found to influence prevention, early diagnosis and safe effective care for diabetes and hypertension among PLWH (Fig. 1), all of which comprised various sub-themes that were barriers and/or facilitators (Table 2). Each theme and sub-theme are described below with supporting quotes provided in Table 3.

**Table 2:** Barriers and facilitators for prevention, early diagnosis and safe effective care from the themes and sub-themes identified

Themes	Sub-themes	Prevention		Early diagnosis		Safe and effective care	
		Barriers	Facilitators	Barriers	Facilitators	Barriers	Facilitators
Organisational and healthcare system factors	Education on lifestyle behaviours		X				
	Counselling on ARV adherence		X				
	Fragmented HIV and NCD services			X		X	
	No protocols on NCD screening			X			
	Lack of access to diagnostic equipment			X			
	Lack of continuity of care for NCDs					X	
Individual factors	HCPs knowledge of NCDs	X			X		
	PLWH knowledge of NCDs	X				X	
	HCPs personal practice				X		X
	Self-monitoring of NCDs						X
Syndemic^a^ factors	Poverty of PLWH	X		X		X	
	HIV stigma			X		X	
	Mental health of PLWH	X					

*ARV* Antiretrovirals, *NCD* Non-communicable disease, *HCPs* Healthcare providers, *PLWH* People living with HIV

^a^Singer and Clair [34] define a syndemic as “a set of intertwined and mutually enhancing epidemics involving disease interactions at the biological level that develop and are sustained in a community/population because of harmful social conditions and injurious social connections”

**Table 3:** Supporting quotes for each theme and sub-theme

Factors that influence prevention of diabetes and hypertension in PLWH		
Organisational and healthcare system factors	Education on lifestyle behaviours	*“in the morning before starting the clinic we give education about diet and we have a nutritional officer in here that helps to plan diet with the client … We also tell them about cigarette smoking and alcohol using and we have people that have already stopped.”* (HIV doctor; P6)
		*“we were also told to do some exercises … exercises like walking … You can go and return by not using car. Other exercises include doing your usual activities.”* (PLWH; P24)
	Counselling on ARV adherence	*“it’s counselling, they all receive this … adherence counselling, they all receive.”* (HIV nurse; P8)
		*“they [CTC HCPs] are the ones that got us to this point because we gave up before when we didn’t have any hope of living, but they gave us education, do this, take this medication, ‘if you adhere to treatment you’ll have life like any other person’.”* (PLWH; P21)
Individual factors	HCP knowledge of NCDs	*“if the patient has hypertension it is easy for them to have diabetes or if they have diabetes it is easy to have hypertension. But on the side of HIV and diabetes or hypertension, from what I know, if the patient’s immunity is low then they can easily get diabetes or hypertension.”* (HIV nurse; P2)
		*“To me I think no there is no association [between HIV, diabetes and hypertension], it just happens coincidently.”* (HIV doctor; P3)
	PLWH knowledge of NCDs	*“we normally receive health education here [at the CTC] and even in the media when we watch TV we may find that a doctor is explaining the risk of getting other infections for people living with HIV … opportunistic infection, for example TB [tuberculosis] and pandemic diseases. If your immunity is weak then you can easily get infected.”* (PLWH; P23)
		*“they [CTC HCPs] never give me that education [on NCDs]. They just give me the HIV education.”* (PLWH; P32)
Syndemic factors	Poverty of PLWH	*“I also advise what is available near their home because it may differ on economic. For me I can eat anything I want but for some patients they can’t eat anything they want.”* (HIV doctor; P3)
		*“one of the things that they told me, first of all is nutrition, good nutrition, to eat a lot of fruits, vegetables, doing exercises, things like that, having a good diet. But you may find some of us we don’t do that because of our economic issues. For example, myself I just eat ugali [porridge made from cornmeal or corn flour], and hard work. Because I cannot afford to take all the things they say I should eat.”* (PLWH; P33)
	Mental health and wellbeing of PLWH	*“sometimes when we take the viral load of patients and you find he is having high viral loads and you’ve been counselling on medication adherence in different sessions and still fail to adhere then you can think that the patient is not stable mentally.”* (HIV nurse; P2)
		*“they told me to avoid overthinking, ‘if someone hurts you, do not take it serious, just take it easy and it will reduce overthinking. You have to take problems as a normal life thing. If you take it very serious, your blood pressure will be high.’.”* (PLWH; P26)
Factors that influence early diagnosis of diabetes and hypertension in PLWH		
Organisational and healthcare system factors	Fragmented HIV and NCD services	*“we may give this medication and believe that when they go to a specific clinic they will be given certain medication. So you may find that the patient does not go to the clinic or they go to the clinic and find there’s a lot of patients waiting on the service and the patient gives up and decides to go home.”* (HIV nurse; P5)
		*“usually they [CTC] refer with a document that says ‘attend so so and so with this diagnosis’.”* (NCD doctor; P11)
	No protocols on NCD screening	*“someone who comes sometimes has symptoms or doesn’t have symptoms. They have [high blood] pressure and they don’t know. If they tell me the symptoms, what I would do, I will provide the CTC care but I will link them with other people in the clinic for hypertension or diabetes.”* (HIV doctor; P1)
		*“we need to have the ability to diagnose early because we may find that the patient has diabetes for a long time or hypertension for a long time without knowing so it is important that we diagnose these patients early.”* (HIV nurse; P2)
	Lack of access to diagnostic equipment	*“we take blood pressure if we have the equipment, if the equipment is not functioning you can just lose them until you suspect that they have hypertension because of their history or their presenting complaints.”* (HIV doctor; P6)
		*“the challenge is on diagnostic tests. You may wish the patient would go for a certain diagnostic test but you may find there is no diagnostic test or you may find the reagent has finished or something like that … it forces you to tell the patient to go outside the facility to look for diagnostic tests.”* (NCD doctor; P20)
Individual factors	HCPs’ knowledge of NCDs	*“You may find the signs and symptoms of diabetes or hypertension, for example he is urinating a lot at night, he is having a lot of thirst, [then] you know exactly that this patient is having diabetes, but to do a confirmation test, they have to go to the lab.”* (HIV nurse; P8)
		*“for example if the client comes and explains ‘I have headache’. For example, yesterday, a client complained with shoulder pain and she just shows us the direction of the pain, that the pain is running across the neck and that is a certain sign that shows you that this needs more investigation so I took her for investigation, they have checked her and they found the blood pressure is high, like 165 over 190.”* (HIV doctor; P6)
	HCPs’ personal practice	*“we must escort them because you can find a client is in line for a long time and if they are going alone to the OPD they have to follow a line again so to avoid that disturbance, we just assist them.”* (HIV doctor; P7)
		*“Then the patient be like ‘ok I have to go home to get money and then come back.’ … remember the blood pressure is already high then he’s telling you he has to go home to find money. So that patient may collapse on the way home. So sometimes us healthcare providers take our money to assist these types of patients.”* (HIV nurse; P2)
Syndemic factors	HIV stigma	*“most of them run to avoid contact with other people. Because we normally tell them when you go to the reception at OPD show them your CTC card [but] you may find they go there and they meet with other patients that are residents of the same place of the HIV patient so the HIV patient may decide to not continue with care at the OPD.”* (HIV doctor; P9)
		*“for big diagnostic tests … it’s just outside the CTC … but there are people who are shy here, they are scared to go from here to the laboratory. They feel that people are seeing them.”* (PLWH; P33)
	Poverty of PLWH	*“The challenge comes when you tell the client ‘now I’m going to take you to another department for the continue of your care’ and some just refuse and some don’t have the money because not all of them have health insurance so the big challenge is money … so when you tell them ‘we are now going to the OPD’, just opening the file is 8,000 TSH. They tell you ‘I don’t have that money’.”* (HIV doctor; P7)
		*“sometimes I want them to take some investigations in the laboratory. And most of them they don’t have money … and if you ask them to take the investigation in another laboratory outside of the hospital, you can see they are disappointed. And even they can tell you that the bus fare they had to borrow from their neighbour. So that is the challenge we are facing.”* (NCD doctor; P17)
Factors that influence safe and effective care		
Organisational and healthcare system factors	Fragmented NCD and HIV services	*“feedback mechanism is one of the big challenges. We find we send a patient to the specific clinic, it would be a clinic for diabetes or hypertension but to give the feedback that ‘we have received this patient’ normally they don’t give feedback. Therefore to receive feedback and to put the notes at the back of the file that this patient also has this problem, they usually don’t do that. Because until the patient comes and explains by himself, that’s when you can know, but apart from that you may find that we never know [about comorbidities].”* (HIV nurse; P5)
		*“so you see they can [suspect] the disease in the CTC but they have to wait for the clinic day in the OPD and by waiting for the clinic day, there can be complications so there is a problem. If there’s a way to improve it, we should start the treatment right after they have been diagnosed.”* (NCD doctor; P11)
	Lack of NCD continuity of care	*“at the hypertension clinic, we meet with different doctors. Today we may meet with this doctor and tomorrow we will meet with a different doctor. But when you meet with different doctors and according to the blood pressure on that particular day they end up changing my medication … they say ‘no you don’t have to use these medications, use these ones’. Now in my opinion, this is what led me to get a stroke, because … I’ve never used a single medication for a long time.”* (PLWH; P25)
		*“clients are used to a certain kind of doctor or healthcare provider but due to scarcity of healthcare workers, they are shifted so you may find one is complaining that I want to be cared for by my doctor or to get a certain room. So it is difficult to convince them to be cared by another doctor or in a different room.”* (NCD nurse; P16)
Individual factors	PLWH knowledge of NCDs	*“they can come from the CTC and take their ARVs and when they come to the NCD clinic [we] give them medication. If you ask them ‘do you have any other problem’ they don’t normally talk. So they just think they are two separate clinics and there’s no need to share their status.”* (NCD doctor; P17)
		*“I don’t know, why should they ask [about HIV]? Like how am I going with my HIV status, how does that concern them? I think they are special for diabetes. Because when you go to the diabetes clinic, what they have to do is check my diabetes status so that I can go home.”* (PLWH; P27)
	HCPs’ personal practice	*“if we find the patient has higher blood pressure for example we normally prefer to start with those patients that have a higher blood pressure compared to those that do not have and we do that because we know these types of patients have to move to another clinic so that is why we prefer to start with them.”* (HIV nurse; P2)
		*“after finishing of taking care on my side for a diabetic patient I do communication with the doctors of CTC either by calling through the phone or escorting the patient … and the CTC doctor will give their advice that is associated with HIV.”* (NCD doctor; P19)
	Self-monitoring of NCDs	*“we normally advise them to take their blood pressure at home and keep a record so that we can know if the prognosis of the client is in line with the medication that we give them.”* (NCD doctor; P11)
		*“I take medication only at night, 2 tablets every night and I test for blood sugar myself at home because I have the diagnostic machine because they say you have to test your blood sugar yourself every day.”* (PLWH; P27)
Syndemic factors	Poverty of PLWH	*“when they are told ‘we don’t have this medication, you have to buy’ the patient comes back to me and says ‘sister, that medication has finished, what should I do, I don’t have money’ so that is one of the challenges. Some of them they don’t have money, even the small amount of 30,000 [TSH] you may encourage the patient to contribute or to pay for a community health fund [insurance]. You find some they don’t have.”* (HIV nurse; P8)
		*“we use medication that is expensive and for many, money is a problem so you may find others that fail to get medication because they don’t have money.”* (NCD doctor; P11)
		*“medication for fever or headache, you’ll find there are no medication and they write for me to buy and I have no money. The diabetic medication, as I have told you I buy when I have money. If I don’t have money, I leave it.”* (PLWH; P32)
	HIV stigma	*“I never told them [OPD staff] I have HIV. They ask but I’m not ready to open up because I know I attend the clinic and I get my medication so there is no need to open up.”* (PLWH; P24)
		*“even if I explain to the diabetes doctor that I have HIV, I don’t think it would help me … if it could be something that was talked about from everyone, maybe we would have more freedom to talk about it.”* (PLWH; P29)

*PLWH* People living with HIV, *N* Nurse, *D* Doctor, *P* Participant, *CTC* Care Treatment Centre, *NCDs* Non-communicable diseases, *OPD* Outpatient department, *TSH* Tanzanian shillings, *ARVs* Antiretrovirals

### Prevention of diabetes and hypertension in PLWH

Both CTC HCPs and PLWH discussed two organisational and healthcare system factors that enable the prevention of diabetes and hypertension: educational sessions on lifestyle behaviours and ARV adherence are provided at the CTC in group and individual settings. However, individual factors regarding HCPs’ and PLWH’s knowledge were identified as barriers: several CTC HCPs thought there were no association between HIV and diabetes or hypertension, or they misunderstood the mechanisms of their association. Further to this, a few PLWH said they were told about diet and exercise by CTC HCPs, but they had not been educated on NCDs prior to their diagnosis, stating that they were only taught about opportunistic infections such as tuberculosis, indicating that the link between lifestyle behaviours and NCD prevention is either not articulated during educational sessions or are poorly understood by PLWH.

Poverty and mental health of PLWH were syndemic factors raised by all groups of participants and were commonly interlinked. To exemplify the former, HCPs and PLWH noted that PLWH often experience difficulties in accessing diverse and healthy foods recommend by HCPs due to direct or indirect costs (i.e. costs of food and costs of travel to buy food for rural inhabitants). Additionally, HCPs and PLWH shared stories of the many stresses PLWH face due to money, food and family issues which often led to PLWH missing their CTC clinic day and thus missing their ARV refills. Many HCPs and PLWH mentioned the burden of ‘overthinking’ about external stresses or PLWH not accepting their HIV diagnosis and how these issues contribute toward poor mental health and wellbeing among PLWH which they said creates a major barrier for ARV adherence.

### Early diagnosis of diabetes and hypertension in PLWH

CTC HCPs were confident they had the adequate knowledge of signs and symptoms for diabetes and hypertension, allowing for timely referral to the OPD for diagnostic tests; an individual factor found to enable early diagnosis. However, an organisational and healthcare system factor found to be a barrier for early diagnosis was the lack of guidelines on NCD screening in PLWH: CTC HCPs said they only refer PLWH for diagnostic tests when certain symptoms are revealed to them which means PLWH with asymptomatic diabetes or hypertension are missed or diagnosed late. Another barrier and healthcare system factor was access to diagnostic equipment. CTC HCPs said they rarely have access to diagnostic equipment, especially for diabetes; though, many said they routinely measure blood pressure when a blood pressure machine is available. Otherwise, for NCDs to be diagnosed, PLWH must attend the OPD and whilst OPD HCPs said they have more equipment, many said blood pressure machines and glucometers were also limited and often broken; as a result, OPD HCPs said it is not unusual to instruct PLWH to find diagnostic tests outside the hospital.

Fragmented HIV and NCD services was another barrier and organisational healthcare system factor raised. No formal referral system exists between the CTC and OPD. CTC HCPs insinuated that they were often unaware if PLWH followed their referral and attended the OPD for diagnostic measurements. HCPs and PLWH said that due to long waiting times, fear of having their HIV status known by OPD staff and patients, and costs (for those without insurance), PLWH opted to not attend the OPD. These latter two points indicate syndemic factors of HIV stigma and poverty, respectively, as barriers for early diagnosis. PLWH are aware that most NCD care costs without insurance; it was strongly expressed from HCPs and PLWH that many PLWH are unable to afford insurance and will not go to the OPD because they lack money for tests (and medication). Despite these challenges, an individual factor related to HCPs’ personal practice was an enabler for early diagnosis: some CTC HCPs said they would personally escort PLWH to the OPD to ensure they receive the care they need, one even said they would give PLWH money they need for tests (and medication).

### Safe and effective diabetes and hypertension care for PLWH

Poverty of PLWH and HIV stigma were also mentioned as barriers for safe and effective diabetes and hypertension care. Many PLWH said they do not regularly attend the OPD due to the cost of diabetes and hypertension medication; some instead buy the medicine outside the hospital where they often must visit multiple places before they find the right medication; others turn to traditional medicine or go without any NCD medication for long periods of time which they said has previously led to complications and admissions to hospital. Such difficulties in receiving NCD medications was said to also cause PLWH stress and negatively impact their mental health and wellbeing. PLWH said they are often reluctant to share their HIV diagnosis with OPD HCPs due to fear of being stigmatised, and CTC HCPs said PLWH do not share their NCD comorbidities with them because they do not understand the importance of doing so (an individual factor related to knowledge). Thus, HCPs said they often do not know about comorbidities nor about other prescriptions, yet drug-drug interactions among PLWH was noted by HCPs as a problem. This lack of oversight of all medications (for safety and adherence control) due to fragmented NCD and HIV services is a key organisational and healthcare system factor that creates a barrier for safe and effective care for diabetes and hypertension among PLWH.

Many PLWH (and one OPD HCP) said NCD continuity of care is a barrier to safe and effective care, an organisational and healthcare system factor. PLWH said they rarely see the same doctor at each appointment and due to poor record keeping, PLWH are often given unnecessary tests and changes to their prescriptions. Again, due to fragmented HIV and NCD care, HCPs and PLWH explained that the clinic days and frequency of follow-up visits between the two are not aligned which causes a burden on PLWH as they must attend the hospital on different days for NCD and HIV care instead of receiving care for all conditions at the same visit. Additionally, one PLWH said they are often told conflicting information from the two clinics. Despite these barriers, HCPs’ personal practice was an individual factor enabling safe and effective care: CTC HCPs from Dodoma Regional Referral Hospital said they operate a triage system whereby PLWH with known NCD comorbidities that are experiencing complications are seen first which enable PLWH to be quickly referred to the OPD for relevant care, and some CTC and OPD HCPs said they call each other to discuss the care and prognosis of PLWH. Furthermore, another individual factor that enables safe and effective care is the ability and willingness of some PLWH to self-monitor diabetes and hypertension through use of a blood pressure machine and blood glucose strips at home, something both HCPs and PLWH mentioned.

## Discussion

This study comprising interviews with key stakeholders (HCPs and PLWH) enabled us to highlight the barriers and facilitators for prevention, early diagnosis and safe effective care of diabetes and hypertension among PLWH within the current model of healthcare delivery. Three main themes were found regarding 1) organisational/healthcare system, 2) individual and 3) syndemic factors. Sub-themes were closely interconnected. Organisational/healthcare system factors comprised the most barriers (four out of six sub-themes) which negatively impacted early diagnosis and safe effective care and limited the positive effects of enabling individual factors. Syndemic factors comprised three sub-themes, all of which were barriers. Prevention, early diagnosis and safe effective care were each negatively impacted by two syndemic factors; thus, syndemic factors were the most prevalent barriers for all three components of care combined. Whilst two organisational/healthcare system factors were facilitators for prevention, barriers related to individual and syndemic factors limited the positive affect of the facilitators.

We found that education on lifestyle behaviours and ARV adherence is provided to PLWH which can prevent diabetes and hypertension among PLWH, but barriers we identified concerning the lack of HCPs’ and PLWH’s knowledge on the relationship between these NCDs and HIV potentially limited the benefits of the educational sessions. Furthermore, PLWH lacked knowledge on how to prevent these NCDs from occurring despite education provided on lifestyle behaviours; they also lacked understanding on how the NCD medications work to control diabetes and hypertension which can negatively affect drug adherence. Our findings corroborate with other qualitative studies regarding PLWH experiences with NCDs [13, 35], indicating that improving health education for both HCPs and PLWH may be warranted. Conversely, CTC HCPs knew about the signs and symptoms of diabetes and hypertension to make a timely referral to the OPD for early diagnosis; however, early diagnosis can only occur if diagnostic equipment is available and operational which was raised as a persistent issue for HCPs in our study. Participants described a layered process required for PLWH to receive a diagnosis which involved multiple opportunities, or weaknesses, for failure, of which fragmented services play a role; this affect closely aligns with the Swiss Cheese Model of accident causation [36]. In turn, missed and late diagnoses of diabetes and hypertension among PLWH is likely a substantial problem within our study setting, as our participants alluded to. To address these insufficient processes required for PLWH to receive a diagnosis of diabetes and hypertension, a solution will need to be developed and agreed through a comprehensive and collaborative approach involving PLWH, HCPs, policy makers, and administration staff and managers.

Despite efforts from individual HCPs and PLWH, fragmented services and a lack of continuity of NCD care reduced the ability for PLWH to receive safe effective care for comorbidities of diabetes and hypertension. Managing diabetes and hypertension among PLWH requires close monitoring of side effects from both ARVs and NCD medications. For instance, Dolutegravir, a first-line ARV with increased use in Africa [37], can negatively interact with metformin if not monitored closely, which can lead to severe lactic acidosis [5]. No oversight of PLWH medications or comorbidities poses a major threat to patient safety [38], potentially increasing the risk of complications, excess morbidity and premature mortality; these outcomes are improved when continuity and linked up care are established [38]. The quality of care is also impacted by which the clinic days at the CTC and OPD are not aligned nor is the number of required follow-up visits; thus, placing a burden on PLWH financially as they must pay for travel and take time away from economic activities for each visit. These findings indicate that the structure of healthcare delivery need to be improved with some level of integration to ensure optimal quality of care and clinical outcomes.

Singer and Clair [34] define a syndemic as “a set of intertwined and mutually enhancing epidemics involving disease interactions at the biological level that develop and are sustained in a community/population because of harmful social conditions and injurious social connections”. Our findings highlight the syndemic of poverty, mental health and stigma and how these adversely interact with the prevention, diagnosis and care of diabetes and hypertension among PLWH. Investigations and medications for diabetes and hypertension were found to be unaffordable for many PLWH in our study, impacting their ability to receive an early diagnosis and effective treatment; this barrier was also found from the 2021 qualitative study conducted in Eastern Tanzania [22]. However, this is not a problem unique to Tanzania; an estimated 39% of people living in LMICs are unable to afford antihypertensive medications or metformin for diabetes control and this is compared to only 1% in high-income countries [39]. Our findings reinforces the urgent call for a streamlined approach to NCD medication procurement across sub-Saharan Africa for reducing their costs and improving their availability [40]; thus, enabling PLWH (and the general population) to effectively control NCDs such as diabetes and hypertension irrespective of their economic position.

HIV-related stigma negatively impacted early diagnosis and safe effective treatment of diabetes and hypertension; this is in line with other qualitative studies with PLWH and comorbidities [41–43]. Concerningly, stigma can also impact ARV adherence and retention in HIV care [41, 44] which may result in reduced opportunities for NCD education and screening for PLWH in addition to the adverse clinical effects of non-adherence to ARVs. Whilst it is unclear whether PLWH in our study experienced enacted or internalised stigma, it is evident from our findings in conjunction with other studies [41–43] that continued efforts for HIV stigma reduction in sub-Saharan Africa is imperative to improve healthcare seeking behaviours, adherence, patient safety and quality of care. Related to ARV adherence and retention in HIV care (thus a barrier for NCD prevention), is poor mental health [45], an issue raised by many of our participants. PLWH have a near two-fold risk for developing depression compared to people without HIV [46] which is likely intertwined with the syndemics of poverty and HIV-related stigma among other factors [46]. The additional burden that PLWH experience to receive a diagnosis, medication and continuity of care for diabetes and hypertension was said to contribute toward poor mental health of PLWH in our study, potentially perpetuating a vicious cycle of non-adherence (to ARVs and NCD medication) and experiences of complications that then fosters or exacerbates poor mental health. Until these syndemic factors are thoroughly addressed alongside contributing organisational and healthcare system factors, diabetes and hypertension care along with HIV care will continue to be adversely impacted.

### Strengths and limitations

We conducted a qualitative study with 36 participants, including HCPs from the OPD, HCPs from the CTC and PLWH with a comorbidity of hypertension and/or diabetes, all groups of which reached saturation. This diverse sample of participants from two large healthcare facilities in the capital city of Dodoma is a major strength of this study. However, there are some limitations to mention. Only PLWH currently receiving HIV care were recruited; PLWH not retained in care may have different or additional barriers to care for diabetes and hypertension. Furthermore, only PLWH with a comorbidity known to the CTC were recruited, but as our study suggests, many diagnoses of diabetes and hypertension are likely unknown to CTC HCPs. The PLWH included in our study may therefore be more comfortable with CTC HCPs or more outspoken about their conditions compared to PLWH whom do not have their comorbidity recorded at the CTC. It is important to note that most of the barriers we present were mentioned by both PLWH and HCPs, strengthening our findings. Although our study was conducted in an urban setting, both healthcare facilities care for PLWH across the Dodoma region which is predominately rural; therefore, our findings are likely transferable to other areas across sub-Saharan Africa where the healthcare delivery of HIV, diabetes and hypertension are not integrated.

## Conclusions

As life expectancy of PLWH continues to rise globally, ensuring healthy aging among PLWH will be an increasing priority. We found organisational/healthcare system, individual and syndemic factors to be interlinked with barriers and facilitators that contribute to the prevention, early diagnosis and safe effective care of diabetes and hypertension among PLWH in our East African setting. Whilst integrated HIV, diabetes and hypertension care is a generally accepted and recommended strategy to reduce barriers of care for PLWH [11], achieving this across sub-Saharan Africa will take time given the uncertainties of how this should be accomplished, and the complexities involved in making large systemic changes to healthcare delivery. Our results indicate that syndemic factors were the most burdensome barriers across the care pathway for PLWH with diabetes and/or hypertension; however organisational/healthcare system had major barriers for early diagnosis and safe effective care. Additionally, barriers related to individual factors were found to limit the enabling factors for prevention. These important and novel findings should inform future large initiatives to integrate care but also provide guidance on specific barriers within the current structure of healthcare delivery that can be targeted through small-scale quick-win interventions to improve the safety and quality of care for diabetes and hypertension among PLWH.

## Data Availability

Data supporting this study are not publicly available due to ongoing unpublished analyses with the data; however, data is available on reasonable request.

## Abbreviations

PLWH: People living with HIV
NCDs: Non-communicable diseases
ARVs: Antiretrovirals
LMICs: Low- and middle-income countries
HCPs: Healthcare providers
CTC: Care treatment centre
OPD: Outpatient department

## References

[1] Jahagirdar D, Walters MK, Novotney A (2021). Global, regional, and national sex-specific burden and control of the HIV epidemic, 1990–2019, for 204 countries and territories: the Global Burden of Diseases Study 2019. Lancet HIV.

[2] Gooden TE, Gardner M, Wang J (2021). Incidence of cardiometabolic diseases in people with and without Human Immunodeficiency Virus in the United Kingdom: a population-based matched cohort study. J Infect Dis.

[3] So-Armah K, Benjamin LA, Bloomfield GS (2020). HIV and cardiovascular disease. Lancet HIV.

[4] Nou E, Lo J, Grinspoon SK (2016). Inflammation, immune activation, and cardiovascular disease in HIV. AIDS.

[5] Noubissi EC, Katte JC, Sobngwi E (2018). Diabetes and HIV. Curr Diab Rep.

[6] Okello S, Amir A, Bloomfield GS (2020). Prevention of cardiovascular disease among people living with HIV in sub-Saharan Africa. Prog Cardiovasc Dis.

[7] Gouda HN, Charlson F, Sorsdahl K (2019). Burden of non-communicable diseases in sub-Saharan Africa, 1990–2017: results from the Global Burden of Disease Study 2017. Lancet Glob Health.

[8] Roy M, Bolton Moore C, Sikazwe I, Holmes CB (2019). A review of differentiated service delivery for HIV treatment: effectiveness, mechanisms, targeting, and scale. Curr HIV/AIDS Rep.

[9] Gay HC, Rao SG, Vaccarino V, Ali MK (2016). Effects of different dietary interventions on blood pressure: systematic review and meta-analysis of randomized controlled trials. Hypertension.

[10] Ettehad D, Emdin CA, Kiran A (2016). Blood pressure lowering for prevention of cardiovascular disease and death: a systematic review and meta-analysis. Lancet.

[11] World Health Organization. Updated recommendations on service delivery for the treatment and care of people living with HIV. Geneva: World Health Organization; 2021. 33999550

[12] Fisher MP, Hamer MK (2020). Qualitative methods in health policy and systems research: a framework for study planning. Qual Health Res.

[13] Yang Z, Zhu Z, Lizarondo L (2021). Experience of chronic noncommunicable disease in people living with HIV: a systematic review and meta-aggregation of qualitative studies. BMC Public Health.

[14] Hing M, Hoffman RM, Seleman J, Chibwana F, Kahn D, Moucheraud C (2019). 'Blood pressure can kill you tomorrow, but HIV gives you time': illness perceptions and treatment experiences among Malawian individuals living with HIV and hypertension. Health Pol Plan.

[15] Morgan SA, Eyles C, Roderick PJ, Adongo PB, Hill AG (2019). Women living with multi-morbidity in the Greater Accra Region of Ghana: a qualitative study guided by the cumulative complexity model. J Biosoc Sci.

[16] The World Bank. World Bank Country and Lending Groups. 2023. https://datahelpdesk.worldbank.org/knowledgebase/articles/906519-world-bank-country-and-lending-groups (accessed 3rd May 2023.

[17] UNAIDS. United Republic of Tanzania. 2021. https://www.unaids.org/en/regionscountries/countries/unitedrepublicoftanzania (accessed 3rd May 2023.

[18] Peck RN, Shedafa R, Kalluvya S (2014). Hypertension, kidney disease, HIV and antiretroviral therapy among Tanzanian adults: a cross-sectional study. BMC Med.

[19] Jeremiah K, Filteau S, Faurholt-Jepsen D (2020). Diabetes prevalence by HbA1c and oral glucose tolerance test among HIV-infected and uninfected Tanzanian adults. PLoS ONE.

[20] Kavishe B, Biraro S, Baisley K (2015). High prevalence of hypertension and of risk factors for non-communicable diseases (NCDs): a population based cross-sectional survey of NCDS and HIV infection in Northwestern Tanzania and Southern Uganda. BMC Med.

[21] Stanifer JW, Cleland CR, Makuka GJ (2016). Prevalence, risk factors, and complications of diabetes in the Kilimanjaro region: a population-based study from Tanzania. PLoS ONE.

[22] Haruna T, Somba M, Siril H (2021). Factors hindering integration of care for non-communicable diseases within HIV care services in Dar es Salaam, Tanzania: The perspectives of health workers and people living with HIV. PLoS ONE.

[23] Pistrang N, Barker C. Varieties of qualitative research: A pragmatic approach to selecting methods. Research designs: Quantitative, qualitative, neuropsychological, and biological American Psychological Association; 2012. https://psycnet.apa.org/record/2011-23864-001.

[24] Kallio H, Pietilä AM, Johnson M, Kangasniemi M (2016). Systematic methodological review: developing a framework for a qualitative semi-structured interview guide. J Adv Nurs.

[25] Sundler AJ, Lindberg E, Nilsson C, Palmér L (2019). Qualitative thematic analysis based on descriptive phenomenology. Nurs Open.

[26] World Population Review. Dodoma Population 2023. 2023. https://worldpopulationreview.com/world-cities/dodoma-population (accessed 19th May 2023.

[27] Palinkas LA, Horwitz SM, Green CA, Wisdom JP, Duan N, Hoagwood K (2015). Purposeful sampling for qualitative data collection and analysis in mixed method implementation research. Adm Policy Ment Health.

[28] Patton MQ (1999). Enhancing the quality and credibility of qualitative analysis. Health Serv Res.

[29] Morse JM (2000). Determining sample size. Qual Health Res.

[30] Cane J, O’Connor D, Michie S (2012). Validation of the theoretical domains framework for use in behaviour change and implementation research. Implement Sci.

[31] Nyangulu W, Mungwira R, Nampota N (2019). Compensation of subjects for participation in biomedical research in resource–limited settings: a discussion of practices in Malawi. BMC Med Ethics.

[32] Gale NK, Heath G, Cameron E, Rashid S, Redwood S (2013). Using the framework method for the analysis of qualitative data in multi-disciplinary health research. BMC Med Res Methodol.

[33] Mattimoe R, Hayden MT, Murphy B, Ballantine J. Approaches to analysis of qualitative research data: A reflection on the manual and technological approaches. Accounting Finance Governance Rev. 2021;27(1):1–15.

[34] Singer M, Clair S (2003). Syndemics and public health: reconceptualizing disease in bio-social context. Med Anthropol Q.

[35] Monroe AK, Rowe TL, Moore RD, Chander G (2013). Medication adherence in HIV-positive patients with diabetes or hypertension: a focus group study. BMC Health Serv Res.

[36] Larouzee J, Le Coze J-C (2020). Good and bad reasons: The Swiss cheese model and its critics. Saf Sci.

[37] Dorward J, Sookrajh Y, Khubone T (2023). Implementation and outcomes of dolutegravir-based first-line antiretroviral therapy for people with HIV in South Africa: a retrospective cohort study. Lancet HIV.

[38] Sheikh A, Dhingra-Kumar N, Kelley E, Kieny MP, Donaldson LJ (2017). The third global patient safety challenge: tackling medication-related harm. Health Organ Bull.

[39] Attaei MW, Khatib R, McKee M (2017). Availability and affordability of blood pressure-lowering medicines and the effect on blood pressure control in high-income, middle-income, and low-income countries: an analysis of the PURE study data. Lancet Public Health.

[40] Siddharthan T, Ramaiya K, Yonga G (2015). Noncommunicable diseases in East Africa: assessing the gaps in care and identifying opportunities for improvement. Health Aff.

[41] Chambers LA, Rueda S, Baker DN (2015). Stigma, HIV and health: a qualitative synthesis. BMC Public Health.

[42] Peer N, de Villiers A, Jonathan D, Kalombo C, Kengne A-P (2020). Care and management of a double burden of chronic diseases: experiences of patients and perceptions of their healthcare providers. PLoS ONE.

[43] Godongwana M, Wet-Billings D, Milovanovic M (2021). The comorbidity of HIV, hypertension and diabetes: a qualitative study exploring the challenges faced by healthcare providers and patients in selected urban and rural health facilities where the ICDM model is implemented in South Africa. BMC Health Serv Res.

[44] Dlamini PS, Wantland D, Makoae LN (2009). HIV stigma and missed medications in HIV-positive people in five African countries. AIDS Patient Care STDS.

[45] Tao J, Vermund SH, Qian HZ (2018). Association between depression and antiretroviral therapy use among people living with HIV: a meta-analysis. AIDS Behav.

[46] Gooden TE, Gardner M, Wang J (2022). The risk of mental illness in people living with HIV in the UK: a propensity score-matched cohort study. Lancet HIV.

